# Metastatic Carcinoid Tumor Presenting As Right Sided Heart Failure

**DOI:** 10.5812/ijem.6927

**Published:** 2013-04-01

**Authors:** Efren Martinez-Quintana, Maria Del Mar Avila-Gonzalez, Laura Suarez-Castellano, Fayna Rodriguez-Gonzalez

**Affiliations:** 1 Cardiology Service, Insular-Materno Infantil University Hospital, Las Palmas de Gran Canaria, Spain; 2 Ophthalmology Service, Dr. Negrin University Hospital, Las Palmas de Gran Canaria, Spain

**Keywords:** Carcinoid, Neuroendocrine, Heart Failure, Tricuspide Valve, Serotonin, Octreotide

## Abstract

Carcinoid tumor is a slow-growing type of neuroendocrine tumor, originating in the enterochromaffin cells and secreting mainly serotonin. The diagnosis is based on clinical symptoms, hormone levels, radiological and nuclear imaging, and histological confirmation. The clinical symptoms are characterized by flushing, diarrhea, abdominal pain, telangiectasia and/or bronchoconstriction. However, most patients have metastatic disease at diagnosis because the clinic goes unnoticed or are ascribed to other abdominal conditions. We report the clinical symptoms, hormone levels, radiological and nuclear imaging, histological diagnosis, treatment and evaluation of a 44-year-old female patient with congestive heart failure secondary to carcinoid heart disease in the context of liver metastases of an ileum carcinoid tumor.

## 1. Introduction

Neuroendocrine tumors (NETs) are a very heterogeneous group arising from the neuroendocrine cells, and include carcinoid, non-carcinoid gastroenteropancreatic tumors (such as insulinoma, gastrinoma and VIPoma (VIP, vasoactive intestinal polypeptide)), catecholamine-secreting tumors (phaeochromocytomas, paragangliomas, ganglioneuromas, ganglioneuroblastomas, sympathoblastoma, neuroblastoma), medullary carcinoma of the thyroid, chromophobe pituitary tumors, small cell lung cancer and Merkel cell tumors.

The different NETs may be divided into functioning and non-functioning tumors. Functioning tumors present clinically with symptoms related to overproduction of hormones and amines such as midgut carcinoids with carcinoid syndrome, gastrinom with Zollinger–Ellison’s syndrome, insulinoma with hypoglycemic symptoms, glucagonoma with glucagonoma syndrome and VIPoma with watery diarrhea–hypokalemia–achlorhydria (WDHA) syndrome. Non-functioning tumors produce and secrete peptides that do not cause any distinct clinical symptom.

The largest group of NETs is the so-called carcinoids, with an average annual incidence rates per 100 000 population of 1.00 and 0.70 for men and women respectively (1). Over two-thirds of carcinoid tumors are found in the gastrointestinal tract and the next most common affected area is the respiratory tract with almost one third of the cases. Carcinoids are thought to arise from the enterochromaffin cells of Kulchitsky found throughout the crypts of Lieberkühn of the gut. Specifically, the term enterochromaffin refers to the ability to stain with chromium or chrome salts, a common feature of serotonin-containing cells (2).

The carcinoid syndrome occurs in approximately 10% of NETs and becomes manifest when vasoactive substances from the tumors, such as serotonin as well as several other chemicals, enter the systemic circulation escaping hepatic degradation. This is the case when NETs metastasize to the liver or they arise for example in the bronchus. The clinical properties is characterized by flushing (63%–94% of patients), diarrhoea (68%–84%), abdominal pain (10%–55%), telangiectasia (25%) and bronchoconstriction (3%–19%) (3). Also, high levels of vasoactive substances released from hepatic metastases causes in 10-20% of patients with carcinoid syndrome the appearance of the endocardium and valvular heart disease.

## 2. Case Report

A 44-year-old female patient, with a history over the last years of facial flush, sweating, palpitations, coughing, breathlessness, intermittent epigastric discomfort, chronic aqueous diarrhea (3 to 4 episodes/day) and weight reduction (approximately ) was sent to our Cardiology Service due to palpitations, progressive dyspnea and lower limb edema. She was previously diagnosed with rosacea, bronchospasm and an irritable bowel syndrome by a dermatologist, a pulmonologist and a gastroenterologist respectively. Previous abdominal ultrasound and upper and lower endoscopy were within normal limits.

Clinical examination showed a hepatomegaly of 4 finger-widths, a soft, depressible and diffusely painful abdomen and lower limb edema. Cardiac auscultation evidenced an holosystolic murmur (III/VI) in the lower left parasternal location that increased with inspiration. Blood analysis showed a mild microcytic anemia (hemoglobin of 10.1 g/dL with a mean corpuscular volume of 72 fL) with an iron deficiency pattern. Serum creatinine, glucose, cholesterol and liver enzymes were within normal limits. Lactate dehydrogenase was 271 U/L (normal range 100–247 U/L).

**Figure 1.**
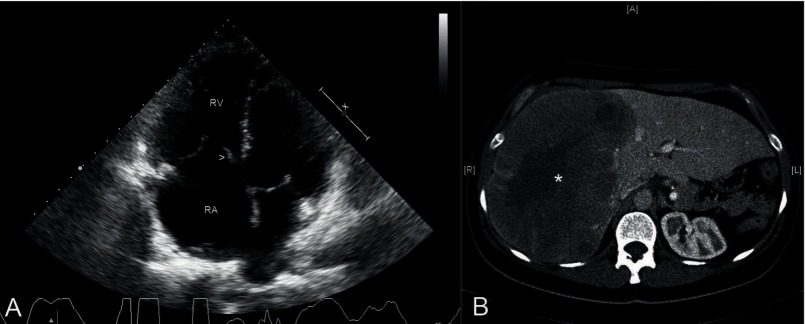
Echocardiography and Abdominal Computed Tomography A: Two-dimensional apical 4-chamber echocardiographic view showing a thickened and fixed tricuspid septal valve (arrow head) that does not allow proper coaptation of the tricuspid valve. RA: right atria, RV: right ventricle. B: Abdominal computed tomography with contrast showing hepatomegaly and a large mass in the right hepatic lobe (asterisk).

Echocardiography showed a normal left ventricle with a normal mitral and aortic valves, a dilated right ventricle with preserved systolic function, a moderate to severe pulmonary regurgitation and a thickened and fixed tricuspid septal valve Figure 1A which resulted in a severe tricuspid regurgitation due to lack of cooptation. During echocardiography, due to the suspicion of carcinoid heart disease, the liver was also examined and a heterogeneous hepatic mass was seen. Abdominal computed tomography (CT) confirmed the existence of a liver mass in the right hepatic lobe of 21 x 12 cms in diameter and a few other isolated liver lesions. Pancreas, adrenal glands, spleen and both kidneys were within normality (Figure 1B).

**Figure 2.**
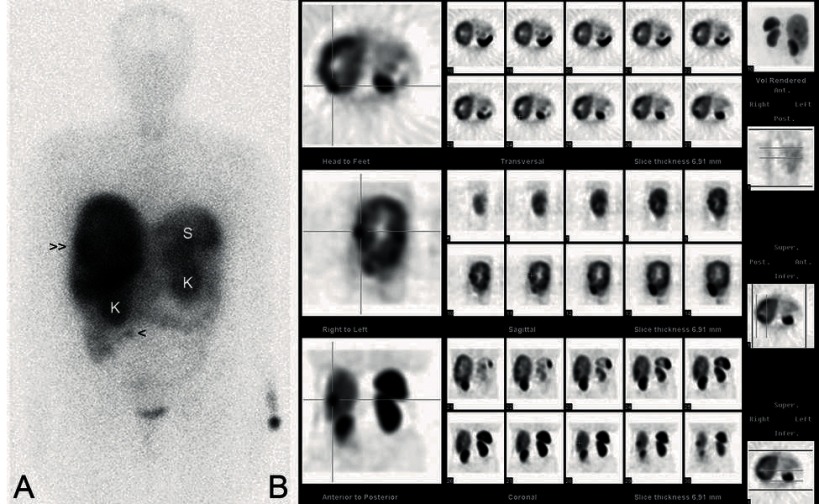
Whole-Body Scintigraphy and Single Photon Emission Computed Tomography (SPECT). Whole-body scintigraphy anterior view (A) and single photon emission computed tomography (SPECT) in the transversal, sagittal and coronal planes (B) after intravenous injection of indium-111-octreotide, showing a large hyperenhanced area that occupies almost the entire right hepatic lobe (double arrowhead) with several focal accumulations of high intensity in both lobes, compatible with an hepatic infiltration by a tumor expressing somatostatin receptors. Also, a focal uptake distal to the inferior pole of the right kidney and apparently localized in the small intestine is seen (arrowhead). Both kidneys uptake the Indium-111-octreotide because it is cleared from the body primarily by renal excretion. Hepatobiliary excretion represents a minor route of elimination, and less than 2% of the injected dose is recovered in feces within three days after injection. Also, ten percent of the radioactivity is excreted as a nonpeptide-bound. Normal distribution of Indium-111-octreotide can be found in the pituitary gland, thyroid gland, liver, spleen, kidneys, urinary bladder and the bowel. L: liver, K: kidney, S: spleen.

Whole-body scintigraphy Figure 2 and single photon emission computed tomography (SPECT) (Figure 2B) after intravenous injection of indium-111-octreotide showed a hepatomegaly of both lobes, highlighting a large and high uptake halo that occupied almost the entire right hepatic lobe with other several liver focal accumulations, consistent with an expressing somatostatin receptor tumor. Also, a focal uptake distal to the inferior pole of the right kidney and apparently localized in the small intestine was seen. This last finding was related to a normal physiological activity or tumor pathology at this level.

The hormonal study showed normal gastrin and vasoactive intestinal peptide levels with high serum serotonin (2358 ng/mL (normal range of 80-450 ng/mL)) and serum chromogranin A (1017.20 ng/mL (normal range of 19-98 ng/mL)) concentrations. 5-hydroxyindoleacetic acid (5-HIAA) in 24 hours urine was 1.3 mg/24 hours(normal range of 0-10).

In the operation room, a giant tumor in the right hepatic lobe with multiple metastases in the left hepatic lobe (intraoperative ultrasound), a tumor in the terminal ileum and pelvic peritoneal carcinomatosis were found. Right hepatectomy, metastasectomy of segments II-III-IV, electrocoagulation of peripheral liver lesions and ileum-cecal resection were done. The terminal ileum histopathological biopsy showed a low mitotic activity neuroendocrine neoplasia in the mucosa of the small intestine which infiltrated all the wall layers and the peripheral fat. Immunohistochemistry revealed the lesion to stain positively for cytokeratin (CK) AE1/AE3, chromogranin and enolase.

The patient underwent chemotherapy with 20 mg octreotide acetate, as an injectable depot formulation every 28 days, which needed to be increased to 30 mg to obtain an acceptable control of the palpitations and the flushing episodes. Ten months later the patient required an additional hepatic surgery for news metastasectomies and radiofrequency.

**Figure 3.**
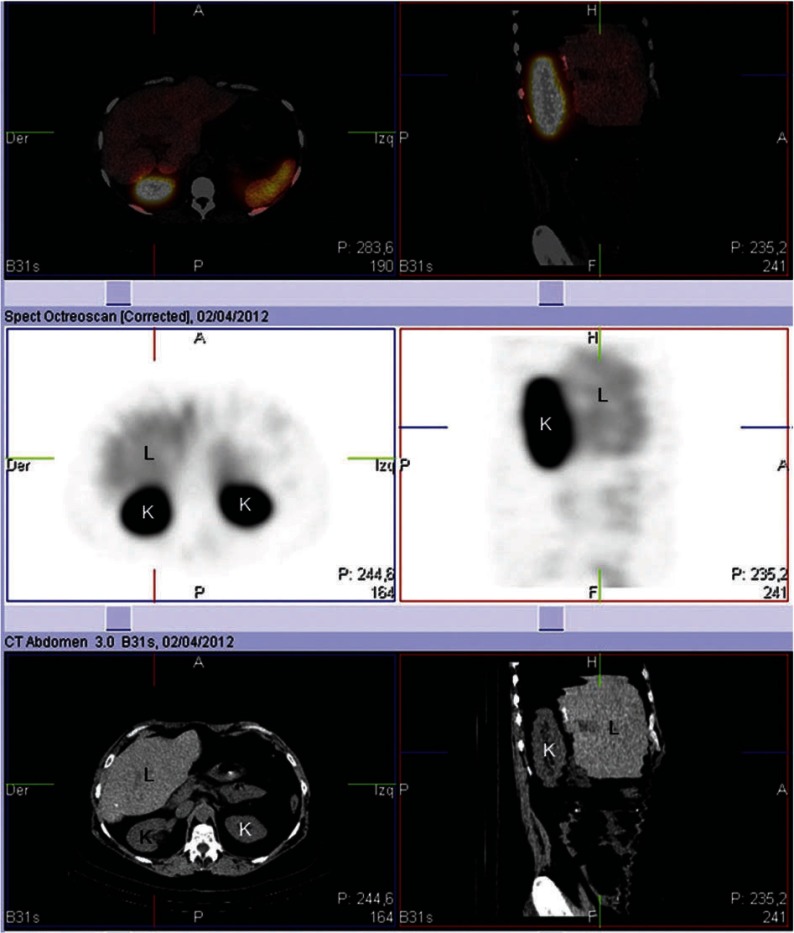
Abdominal Computed Tomography, Whole-Body Scintigraphy and Hybrid Study Abdominal computed tomography (CT) (lower images), whole-body scintigraphy (central images) and hybrid study [single photon emission computed tomography (SPECT)-(CT)] (upper images) with indium-111-octreotide showing a typical distribution of the radiotracer with preferential visualization of the liver, the spleen and the renal system without pathological accumulation activity. L: liver, K: kidney.

Actually, and 22 months after the initial liver surgery, the patient is in a II/IV functional class of the New York Heart Association, has an appropriate control of the gastrointestinal symptoms and has no data on biochemical recurrence (serum serotonin of 243 ng/mL and serum chromogranin A of 20.9 ng/mL). The hybrid study (SPECT-CT) with indium-111-octreotide shows a typical distribution of the radiotracer with preferential visualization of the liver, the spleen and the renal excretory system without pathological accumulation activity (Figure 3). Since the patient has recently had two abdominal surgeries plus chemotherapy and is actually in an acceptable functional class, tricuspid valve repair or the need of valvular replacement has been postponed.

## 3. Discussion

The incidence of small intestine cancer has increased over the past several decades with a four-fold increase in carcinoid tumors, less dramatic rises for adenocarcinoma and lymphoma and stable sarcoma rates. Very little is known about its etiology. An increased risk has been noted for individuals with Crohn’s disease, celiac disease, adenoma, familial adenomatous polyposis and Peutz-Jeghers syndrome. Also, several behavioral risk factors including consumption of red or smoked meat, saturated fat, obesity and smoking have been suggested (4).

NETs are slow growing and may be present for years without overt symptoms, thus escaping attention, becoming manifest after metastases, frequently to regional lymph nodes, liver, and, less commonly to the bone, has occurred. In fact, most patients have metastatic disease at diagnosis, with regional or distant metastasis observed in 50% of patients (5).

The diagnosis is based on clinical symptoms, hormone levels, radiological and nuclear imaging, and histological confirmation. During the early stages, vague abdominal pain goes undiagnosed and invariably is ascribed to irritable bowel or spastic colon as occurred in our patient and frequently, tumor localization may be extremely difficult because barium swallow and follow-up examination of the intestine may only occasionally show the tumor. However, capsule video endoscopy can help locate it and laparotomy is often the only definitive way to determine its presence

Recent studies have suggested that chromogranin A should be the primary biomarker used for the diagnosis of NETs as levels correlate with tumor burden. Also, 5-HIAA, a breakdown component and the end product of serotonin metabolism, is used to identify certain types of functioning NETs having a sensitivity of 73% and a specificity of 100% in predicting the presence of a midgut NETs. However, in some cases, the tumors are small and don't release enough serotonin for a positive test result. In other cases, the tumors do not make much serotonin, but they do make its precursor (6).

For localization of both primary lesions and metastasis, the initial imaging method is Octreoscan, where indium-111 labelled analogues are used in for detecting tumors expressing receptors having an overall sensitivity of 80 to 90% and has the advantage of scanning the entire body (7).

Carcinoid heart disease is a well-known complication of long-lasting exposure to high levels of serotonin, occurring in 20-70% of the patients with metastatic well-differentiated NETs (8, 9). Carcinoid heart disease is characterized by plaque-like, fibrous thickening of the endocardium, classically on the right side of the heart (10), and tricuspid and pulmonary valves (11), being right-sided carcinoid heart disease associated with substantial morbidity and mortality (12).

The primary treatment goal for patients with NETs is curative, with symptom control and the limitation of tumor progression as secondary goals. Surgery is the only possible curative approach and so represents the traditional first-line therapy. However, as most patients with NETs are diagnosed once metastases have occurred, curative surgery is generally not possible. Patients therefore require chronic postoperative medical management with the aim of relieving symptoms and, in recent years, suppressing tumor growth and spread. Octreotide, a somatostatin analogue which decreases the secretion of serotonin by the tumor and, secondarily, decreases the breakdown product of serotonin (5-HIAA) can improve the symptoms of carcinoid syndrome and stabilize the tumor growth in many patients (13).

The prognosis for patients with metastatic carcinoid tumors has improved during the last decade. According to the data from US SEER (United States Surveillance Epidemiology and End Results) data for the period 1992 to 2005, the 5 years relative survival was 80.7% for neuroendocrine (primarily carcinoid) cancers (2). However, five-year survival after resection has remained relatively unchanged over the last 20 years after adjusting for changes in patient demographics, tumor characteristics, and treatment approaches (14) and due to longer survival times, complications, such as carcinoid heart disease, and new metastatic patterns, like skin and bone metastases, may become more important features of carcinoid disease.

In patients with severe cardiac involvement and well-controlled systemic disease, cardiac surgery has been recognized as the only effective treatment option. The timing of tricuspid surgical intervention remains controversial, mostly due to the limited data available and their heterogeneous nature. As a general principle—if technically possible—valve repair is preferable to valve replacement and surgery should be carried out early enough to avoid irreversible right ventricular dysfunction. The European Society of Cardiology Guidelines list tricuspid valve surgery as a Class I indication in the case of symptomatic patients with severe isolated primary tricuspid regurgitation without severe right ventricular dysfunction and a Class IIa indicated in the case of asymptomatic or mildly symptomatic patients with severe isolated primary tricuspid regurgitation and progressive right ventricular dilatation or deterioration of right ventricular function (15).

Increasing tricuspid regurgitation severity is associated with worse survival regardless of the left ventricular ejection fraction or pulmonary artery pressure and is associated with a poor prognosis, independent of age, biventricular systolic function, right ventricular size, and dilation of the inferior vena cava (16). Though patients with severe tricuspid regurgitation respond well to diuretic therapy, delaying surgery is likely to result in irreversible right ventricular damage, organ failure, and poor results of late surgical intervention. In fact, tricuspid valve surgery may not only be beneficial in terms of symptom relief, but may also contribute to the improved survival observed over the past two decades in patients with carcinoid heart disease. For this reason, early diagnosis and early surgical treatment in appropriately selected patients may provide the best results (17).
